# Gastrointestinal bleeding is associated with higher in-hospital mortality, longer length of stay and higher cost in patients with in-hospital cardiac arrest

**DOI:** 10.1016/j.resplu.2021.100150

**Published:** 2021-07-08

**Authors:** Guangchen Zou, Gin-Yi Lee, Yee Hui Yeo, Tien-Chan Hsieh, Kaiqing Lin

**Affiliations:** a Danbury Hospital, Danbury, CT, USA; b University of Vermont, Burlington, VT, USA; c Cedars-Sinai Medical Center, Los Angeles, CA, USA

**Keywords:** cardiac arrest, gastrointestinal bleeding

## Abstract

**Background:**

In-hospital cardiac arrest (IHCA) carries a high mortality and significant morbidity in survivors. Gastrointestinal bleeding (GIB) can complicate cardiac arrests. We aim to study the association between GIB and the in-hospital outcomes of patients with IHCA.

**Methods and results:**

The National Inpatient Sample 2016–2018 databases were used. IHCA were identified using ICD-10-PCS code for cardiopulmonary resuscitation. Other diagnoses including GIB were identified using ICD-10-CM codes. Multivariate logistic regression was used to study the effect of GIB on in-hospital mortality. Gamma regression with log link was used to determine the effect of GIB on length of stay and cost of admission. In patients with IHCA, GIB as a secondary diagnosis is associated with an increased in hospital mortality (unadjusted 74.2% vs 68.3%, adjusted OR 1.17, 95% confidence interval [CI] 1.09–1.25, p < 0.001), longer length of stay (unadjusted median 16 vs 10 days, IQR 9–27 vs 5–17 days, exponentiated coefficient 1.45, 95% CI 1.36–1.54, p < 0.001 for survivors; unadjusted median 4 vs 3 days, IQR 1–10 vs 1–7 days, exponentiated coefficient 1.27, 95% CI 1.22–1.34, p < 0.001 for patients who died in hospital), and higher cost for hospital stay (unadjusted median $226065 vs $151459, IQR $117551–434003 vs $76197–287846, exponentiated coefficient 1.40, 95% CI 1.32–1.49, p < 0.001 for survivors; unadjusted median $87996 vs $77056, IQR $42566–186677 vs $34066–149009, exponentiated coefficient 1.26, 95% CI 1.20–1.32, p < 0.001 for patients who died in hospital) adjusted for baseline characteristics and other comorbidities.

**Conclusions:**

In patients with IHCA, GIB as a secondary diagnosis is associated with a higher in-hospital mortality, longer length of stay and higher cost for the admission.

## Introduction

Over 290,000 adults in the United States suffer in-hospital cardiac arrest (IHCA) each year which carries a high mortality and can lead to significant morbidity in survivors.^1^ Outcomes of in-hospital cardiac arrest depend on many factors including the cause of the cardiac arrest^2^ and arrest rhythm.^3^ While studies have found a higher Charlson Comorbidity Index (CCI) or the presence of certain comorbidities such as diabetes and renal disease is associated with a higher mortality in out-of-hospital cardiac arrests,^4^, ^5^ few studies have looked at the effects of comorbidities on the outcomes of IHCAs. Here we study the association between the presence of a secondary diagnosis gastrointestinal bleeding (GIB) and the outcomes of IHCA using the National Inpatient Sample (NIS).

The NIS is developed by the Agency for Healthcare Research and Quality (AHRQ). It is the largest publicly available all-payer inpatient healthcare database in the United States and contains data from more than 7 million hospital stays each year.

## Methods

The National Inpatient Sample 2016–2018 databases were used. We identified IHCA using ICD-10-PCS code for cardiopulmonary resuscitation. GIB and severe sepsis were identified using ICD-10-CM codes. A shockable arrest rhythm was identified with the presence of ICD-10-CM codes for ventricular tachycardia or ventricular fibrillation. Other comorbidities were identified, and Charlson Comorbidity Index and Elixhauser Comorbidity Index were calculated using coding algorithms developed by Quan et al.^6^ with codes from University of Manitoba.^7^ Interventions such as targeted temperature management and mechanical ventilation were identified using ICD-10-PCS codes. The ICD-10-CM and ICD-10-PCS codes used are listed in Supplementary Table 1. Patients with a primary diagnosis of GIB were excluded.

Data processing and statistical analysis were done using SAS 9.4 (SAS Institute Inc., Cary, NC, USA) and RStudio 1.3.1093 (RStudio, PBC, Boston, MA, USA). Multivariate logistic regression was used to study the effect of GI bleeding on in-hospital mortality, controlling for age (in decades), gender, race, primary expected payer, shockable arrest rhythm, targeted temperature management, other acute conditions or comorbidities including severe sepsis and individual groups of CCI except for peptic ulcer disease. Generalized linear regression using a gamma distribution with log-link was used to determine the effect of GIB on length of stay and the cost of the index admission controlling for the above factors stratified by if death occurred during hospital stay. Cost was adjusted for inflation to 2021 US dollars. P value of <0.01 were considered significant. To study the effect of targeted temperature management on GIB in patients with IHCA, logistic regression was used adjusting for age (in decades), gender, race, primary expected payer, shockable arrest rhythm, comorbidities including severe sepsis and individual groups of Charlson Comorbidity Index including peptic ulcer disease.

## Results

**Fig. 1:**
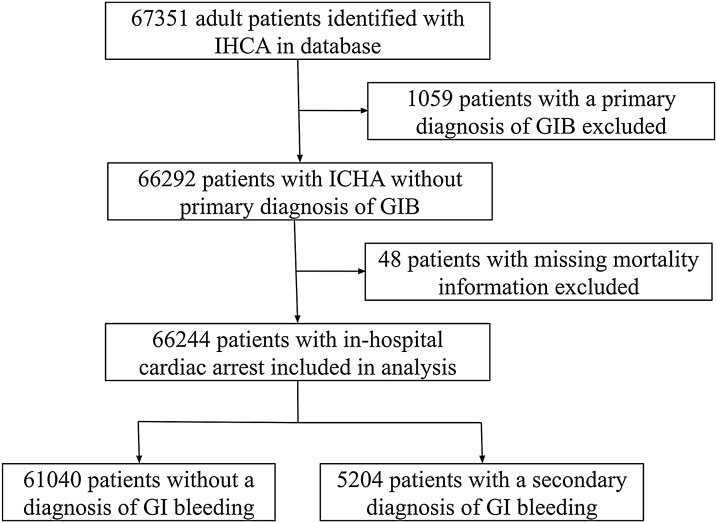
Study cohort.

67351 adult patients with IHCA were identified in the databases. 1059 patients have a primary diagnosis of GIB and was thus excluded. 48 patients with missing mortality data were excluded. 66244 adult patients with IHCA were included in the analysis (Fig. 1). Among them, 5204 (7.86%) patients have a second diagnosis of GIB. The baseline characteristics of the IHCA patients with and without cardiac arrest were listed in Table 1. Unadjusted for other factors, IHCA patients with a secondary diagnosis of GIB more likely to be of non-Caucasian race (42.2% vs 38.3%, p < 0.001) and less likely to have a shockable arrest rhythm (25.3% vs 28.2%, p < 0.001). Similar percentage of patients in both groups underwent targeted temperature management (1.9% vs 1.8%, p = 0.937). Patients with a secondary diagnosis of GIB have a higher Charlson Comorbidity Index (CCI) when peptic ulcer disease (PUD) is included in the CCI (mean ± SD 2.70 ± 1.57 vs 2.53 ± 1.59, p < 0.001). The difference in CCI is smaller when PUD is excluded from the CCI (2.59 ± 1.53 vs 2.52 ± 1.58, p = 0.004).

**Table 1:** Baseline characteristics of patients with in-hospital cardiac arrest with and without a secondary diagnosis of GI bleeding.

Variables, no.		No GI bleeding	GI bleeding	P value
		(N = 61040)	(N = 5204)	
Death in hospital (%)		41679 (68.3)	3860 (74.2)	<0.001
Age (median [IQR])		67.00 [57.00, 77.00]	67.00 [57.00, 76.00]	0.001
Sex (%)	Male	34948 (57.3)	3056 (58.7)	0.043
	Female	26078 (42.7)	2148 (41.3)	
Race (%)	Caucasian	36384 (61.7)	2900 (57.8)	<0.001
	African American	12642 (21.4)	1154 (23.0)	
	Hispanic	5928 (10.1)	537 (10.7)	
	Asian or Pacific Islander	1809 (3.1)	228 (4.5)	
	Native American	330 (0.6)	33 (0.7)	
	Other	1876 (3.2)	163 (3.3)	
Primary payer (%)	Medicare	38269 (62.8)	3148 (60.6)	<0.001
	Medicaid	7950 (13.0)	807 (15.5)	
	Private insurance	10629 (17.4)	858 (16.5)	
	Self-pay	2505 (4.1)	232 (4.5)	
	No charge	140 (0.2)	14 (0.3)	
	Other	1475 (2.4)	132 (2.5)	
Hospital (rural vs urban) (%)	Rural	3359 (5.5)	248 (4.8)	0.042
	Urban non-teaching	13901 (22.8)	1159 (22.3)	
	Urban teaching	43780 (71.7)	3797 (73.0)	
Hospital bed-size (%)	Small	9525 (15.6)	817 (15.7)	0.666
	Medium	18026 (29.5)	1506 (28.9)	
	Large	33489 (54.9)	2881 (55.4)	
Shockable arrest rhythm (%)		17209 (28.2)	1317 (25.3)	<0.001
Targeted temperature management (%)		1122 (1.8)	97 (1.9)	0.937
Mechanical ventilation (%)		43069 (70.6)	4133 (79.4)	<0.001
Severe sepsis (%)		15847 (26.0)	1960 (37.7)	<0.001
Myocardial infarction (%)		17817 (29.2)	1401 (26.9)	0.001
Heart failure (%)		28664 (47.0)	2204 (42.4)	<0.001
Cerebrovascular disease (%)		6696 (11.0)	584 (11.2)	0.592
Chronic lung disease (%)		17346 (28.4)	1401 (26.9)	0.022
Peptic ulcer disease (%)		540 (0.9)	598 (11.5)	<0.001
Liver disease without cirrhosis (%)		3699 (6.1)	701 (13.5)	<0.001
Diabetes with complications (%)		12936 (21.2)	1020 (19.6)	0.007
Kidney disease (%)		21573 (35.3)	1871 (36.0)	0.385
Cancer (%)		6221 (10.2)	668 (12.8)	<0.001
Liver disease with cirrhosis (%)		1820 (3.0)	557 (10.7)	<0.001
Metastatic cancer (%)		2943 (4.8)	315 (6.1)	<0.001
Charlson comorbidity index (mean (SD))		2.53 (1.59)	2.70 (1.57)	<0.001
Charlson comorbidity index without peptic ulcer disease (mean (SD))		2.52 (1.58)	2.59 (1.53)	0.004
Elixhauser comorbidity index (mean (SD))		5.64 (2.31)	6.07 (2.16)	<0.001
Length of stay (median [IQR])		4.00 [1.00, 11.00]	6.00 [2.00, 15.00]	<0.001

IQR = interquartile range; SD = standard deviation

**Fig. 2:**
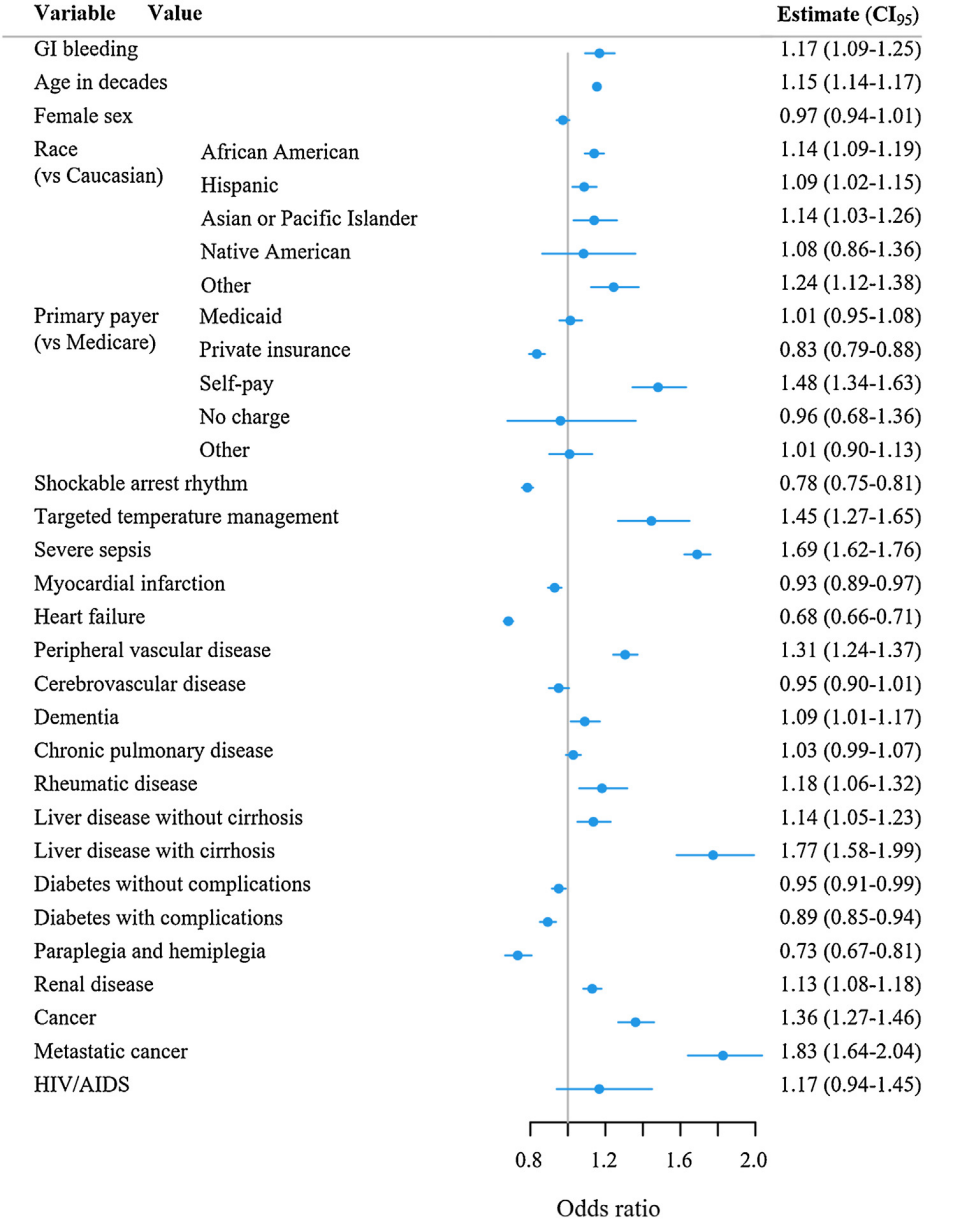
Forest plot of effects of different factors on in-hospital mortality of patients with IHCA. Effects expressed using odds ratios from logistic regression.

In adult patients with IHCA, patients with a secondary diagnosis of GIB have a higher in-hospital mortality adjusted for age (in decades), sex, race, arrest rhythm, acute conditions or comorbidities including severe sepsis and individual groups of CCI excluding PUD, and if patient underwent targeted temperature management (unadjusted 74.2% vs 68.3%, adjusted OR 1.17, 95% CI 1.09-1.25, p < 0.001) (Fig. 2). Other factors associated with an increased in-hospital mortality include non-shockable arrest rhythm (OR 1.28, 95% 1.23–1.33, p < 0.001), older age (in decades, OR 1.16, 95% CI 1.14–1.17, p < 0.001), a diagnosis of metastatic cancer (OR 1.83, 95% CI 1.64–2.04, p < 0.001) or any cancer (OR 1.36, 95% CI 1.27–1.46), liver disease with cirrhosis (OR 1.77, 95% CI 1.58–1.99, p < 0.001) or liver disease without cirrhosis (OR 1.14, 95% CI 1.05–1.23, p = 0.001), severe sepsis (OR 1.69, 95% CI 1.62–1.76, p < 0.001), peripheral vascular disease (OR 1.31, 95% CI 1.24–1.37, p < 0.001), kidney disease (OR 1.13, 95% CI 1.08–1.18, p < 0.001), and rheumatic/connective tissue disease (OR 1.18, 95% CI 1.06–1.32, p = 0.003). Among expected primary payers, self-pay is associated with higher adjusted in-hospital mortality (OR 1.48, 95% CI 1.35–1.63, p < 0.001) while private insurance is associated with lower adjusted in-hospital mortality (OR 0.83, 95% CI 0.79–0.88, p < 0.001) compared with Medicare. Among different races, compared with Caucasians, a race of African American, Hispanic, or Asian and Pacific Islanders are all associated with a higher adjusted in-hospital mortality (OR 1.14, 95% 1.09–1.19 for African American, p < 0.001; OR 1.09, 95% CI 1.02–1.15 for Hispanic, p = 0.006; OR 1.14, 95% CI 1.03–1.26 for Asian and pacific islander, p = 0.011).

**Fig. 3:**
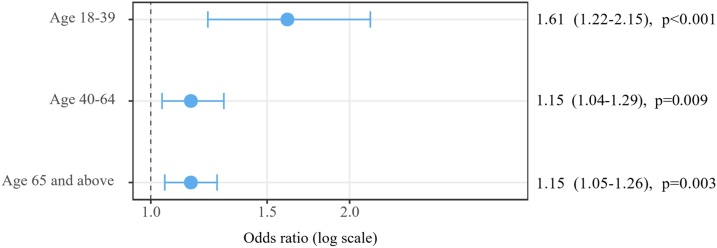
Forest plot of the adjusted effect of a secondary diagnosis of GIB on in-hospital mortality in patients with IHCA in different age groups. Result of multivariate logistic regression. Expressed as OR (95% CI).

Among different age groups (age 18-39, age 40-64, and age 65 and above), GIB as a secondary diagnosis is associated with an increased in-hospital mortality in all age groups, with the highest OR in patients between age 18 and 39 (Fig. 3).

**Fig. 4:**
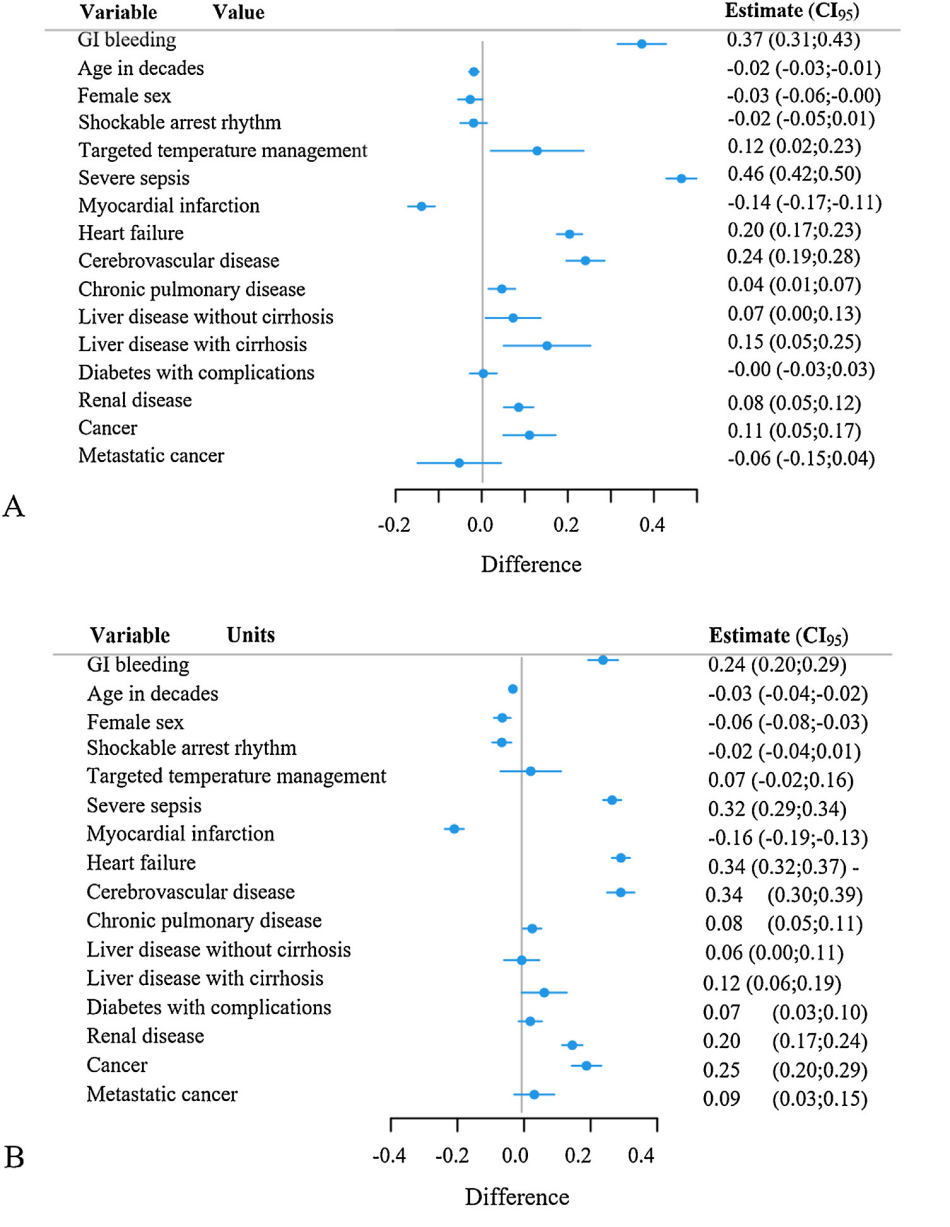
Forest plot of effects of different factors on length of stay. Analysis using gamma regression with log link in patients who survived (A) or died in hospital (B).

In patients with IHCA, a secondary diagnosis of GIB is associated with a longer length of say both in patients who survived the hospital stay (unadjusted median 16 vs 10 days, IQR 9–27 vs 5–17 days, exponentiated coefficient 1.45, 95% CI 1.36–1.54, p < 0.001) and in patient who died in hospital (unadjusted median 4 vs 3 days, IQR 1–10 vs 1–7 days, exponentiated coefficient 1.27, 95% CI 1.22–1.34, p < 0.001), adjusted for age (in decades), sex, race, arrest rhythm, comorbidities including individual groups of CCI excluding PUD, severe sepsis, and if patient underwent targeted temperature management (Fig. 4). A secondary diagnosis of GIB is also associated with a higher total charge for the hospital stay adjusted for inflation into 2021 US dollars, both in patients who survived the hospital stay (unadjusted median $226065 vs $151459, IQR $117551–434003 vs $76197–287846, exponentiated coefficient 1.40, 95% CI 1.32–1.49, p < 0.001) and in patients who died in hospital (unadjusted median $87996 vs $77056, IQR $42566–186677 vs $34066–149009, exponentiated coefficient 1.26, 95% CI 1.20–1.32, p < 0.001), adjusted for the same factors.

The use of targeted temperature therapy was not associated with an increased adjusted risk of having GIB adjusted for age (in decades), gender, race, primary expected payer, shockable arrest rhythm, comorbidities including severe sepsis and individual groups of Charlson Comorbidity Index including peptic ulcer disease (Supplementary Table 2). Factors associated with an increased risk of GIB include a diagnosis of PUD (OR 13.9, 95% CI 12.27–15.78, p < 0.001), invasive mechanical ventilation (OR 1.43, 95% CI 1.32–1.54, p < 0.001), a diagnosis of severe sepsis (OR 1.45, 95% CI 1.36–1.55, p < 0.001), a diagnosis of liver disease with cirrhosis (OR 2.74, 95% CI 2.43–3.10, p < 0.001) or without cirrhosis (OR 1.60, 95% CI 1.44–1.78, p < 0.001), a diagnosis of cancer (OR 1.23, 95% CI 1.10–1.36, p < 0.001) or a race of Asian or Pacific Islander or African American vs Caucasian (OR 1.42, 95% CI 1.22–1.65 for Asian or Pacific Islander, p < 0.001; OR 1.11, 95% CI 1.03–1.20 for African American, p = 0.006).

## Discussion

IHCA carries a high mortality but survival to discharge had improved over the past 2 decades.^8^ The effects of comorbidities on the outcomes and healthcare resource utilization of IHCA have not been well studied. Our study showed in patients with IHCA, GIB as a secondary diagnosis is associated with an increased in-hospital mortality, longer length of stay and higher cost of hospital stay.

The study has important limitations. Coding errors or omissions may be present in this administrative database and information such as medication use, alcohol use or previous history of GIB is not available from this data base. Other factors that will affect outcomes include the direct cause of the cardiac arrest and time to initiation of CPR are not available. It is also impossible to ascertain from the database whether GIB occurred before or after IHCA. GIB, especially esophageal variceal bleeding can also be the cause of ICHA and is associated with worse outcomes.^2^ The effects of this on our result should be limited because patients with a primary diagnosis of GIB were excluded from our analysis and after this exclusion no patient carries a diagnosis of esophageal varices with bleeding. Most importantly, GIB may also be a maker of patients who were sicker or more complicated in ways that were not captured by comorbidity indices and other factors we included in the analysis. Despite these limitations, the increased mortality and healthcare resource utilization associated with a secondary diagnosis of GIB in this large real-world cohort is notable.

GIB is an important complication of hospitalization^9^ especially in critically ill patients.^10^ As a complication, it is associated with worse outcomes in may acute illnesses such as sepsis and stroke.^11^, ^12^, ^13^ Here we showed its association with worse outcomes in patients with IHCA. Patient with IHCA may be particularly at high risk for GIB given they are often critically ill and require invasive mechanical ventilation (70.1% in our sample). Our study failed to show any association between targeted temperature management and GIB in IHCA patients. This is consistent with previous studies.^14^, ^15^ The association between GIB and increased mortality holds true in all age groups, but the OR was highest in the age 18–40 group. This is possibly related to the different disease spectrum and direct causes of IHCA in this age group.

In summary, GIB is an important comorbidity for patients with IHCA and is associated with increased mortality, longer length of stay and high hospital charge. It is important for clinicians to be vigilant about GIB when taking care of patients of IHCA. Prevention strategies such as acid suppression with a proton pumps inhibitor or histamine-2 receptor antagonist and/or early institution of enteral nutrition should be considered for appropriate patients at high risk for GIB.^10^

## Funding

National Inpatient Sample Databases were purchased using educational stipends from Nuvance Health Danbury Hospital.

## Declaration of interests

The authors declare that they have no known competing financial interests or personal relationships that could have appeared to influence the work reported in this paper.
